# Effects of Two Different Rotary Files on Crack Formation in Dentin: A Scanning Electron Microscopy (SEM) Study

**DOI:** 10.7759/cureus.82349

**Published:** 2025-04-16

**Authors:** Ataul Hafeez Imran

**Affiliations:** 1 Conservative Dentistry and Endodontics, Noor Hospital, Qadian, IND

**Keywords:** dentinal cracks, mtwo, protaper next, root canal preparation, rotary file systems

## Abstract

Introduction

Root canal preparation is a critical step in endodontic success, with rotary file systems playing a key role in shaping and cleaning the root canal system. This study aimed to compare the effects of ProTaper Next (Dentsply Maillefer, Ballaigues, Switzerland) and Mtwo nickel-titanium (Ni-Ti) (VDW, Munich, Germany) rotary file systems on dentinal crack formation using a scanning electron microscope (SEM) (Zeiss Evo 18; Carl Zeiss AG, Oberkochen, Germany).

Methods

Fifteen patients requiring bilateral extraction of single-rooted mandibular premolars for orthodontic reasons were selected. Extracted teeth were divided into two groups: group 1 (ProTaper Next) and group 2 (Mtwo). After standardized root canal preparation, the specimens were sectioned at 3, 6, and 9 mm from the apex and examined under SEM for crack formation.

Results

Both file systems induced dentinal cracks post-instrumentation. ProTaper Next files resulted in fewer cracks than Mtwo, but the difference was not statistically significant (p > 0.05). Cracks were more prevalent in the coronal third for both groups. The Mtwo system caused more cracks in the coronal and middle thirds, though this difference remained non-significant (p > 0.05). A statistically significant difference (p < 0.05) was observed in the apical third, where the Mtwo files induced more cracks than ProTaper Next.

Conclusion

Both ProTaper Next and Mtwo rotary files contribute to dentinal crack formation, with ProTaper Next causing fewer cracks, particularly in the apical third. These findings underscore the clinical importance of file selection in minimizing dentinal damage and reducing the risk of root fractures during endodontic treatment.

## Introduction

Endodontic treatment, particularly root canal therapy, is a complex procedure aimed at preserving the natural dentition by eliminating infections and preventing reinfection within the root canal system [1]. The success of root canal therapy depends on thorough cleaning, shaping, and three-dimensional obturation to ensure complete removal of microbial biofilms, necrotic tissue, and debris [2]. Biomechanical preparation is a critical phase in this process, as it facilitates bacterial elimination while shaping the canal for effective obturation. The instrumentation used during this phase plays a significant role in determining canal cleanliness and the preservation of root dentin integrity.

Dentin, the primary structural component of a tooth, is a mineralized tissue composed of approximately 60% inorganic material (primarily carbonated apatite), 30% organic content (predominantly type I collagen), and 10% water [3]. The inorganic fraction contributes to the stiffness and compressive strength of the tooth, enabling it to withstand masticatory forces, while the organic component enhances tensile strength, resistance to crack propagation, and overall durability. Water content plays a vital role in maintaining dentin’s viscoelastic properties and stress distribution. Any alteration to dentin integrity during biomechanical preparation can compromise these properties, potentially leading to microcrack formation and vertical root fractures, which may jeopardize long-term treatment success [4].

Traditionally, stainless steel hand files were used for root canal preparation. However, these instruments had inherent limitations, particularly in curved canals, where they often caused procedural errors such as ledging, apical transportation, and perforation. The introduction of nickel-titanium (Ni-Ti) rotary instruments revolutionized endodontic treatment by improving canal cleanliness, reducing procedural mishaps, and maintaining the canal’s natural curvature. Ni-Ti instruments possess superior flexibility and cutting efficiency, allowing for more predictable shaping while minimizing unnecessary dentin removal [5].

Despite these advancements, rotary instrumentation generates stress concentrations along dentinal walls, potentially inducing microcracks or craze lines. These structural defects may increase the risk of vertical root fractures, which are a major complication often leading to tooth loss. Several factors influence stress distribution in the root canal, including the instrument’s taper, cross-sectional shape, flute design, and cutting efficiency. Studies suggest that circular canal cross sections minimize stress concentrations and promote uniform stress distribution, thereby reducing the likelihood of crack formation. Additionally, preserving dentin thickness, particularly in vulnerable areas, is crucial to maintaining tooth strength. The application of lubricants and appropriate instrument selection can help mitigate excessive stress during root canal preparation [6].

The development of Ni-Ti rotary instrumentation has undergone several generations of refinement to enhance performance and safety [7]. 

First-generation Ni-Ti rotary files

These files featured passive cutting radial lands and fixed tapers (4% or 6%), requiring multiple instruments to achieve proper canal shaping.

Second-generation Ni-Ti rotary files

Introduced in 2001, these instruments incorporated active cutting edges, reducing the number of files required for preparation. A major innovation in this category was the ProTaper system, which featured progressive and regressive tapers on a single file, enabling efficient and conservative canal preparation.

Third-generation Ni-Ti rotary files

This phase focused on improving metallurgical properties, leading to the development of heat-treated Ni-Ti alloys. Systems such as Twisted File, HyFlex, and GT Vortex demonstrated enhanced cyclic fatigue resistance and flexibility, reducing the risk of file separation. These properties allowed for safer and more predictable instrumentation, particularly in complex root canal anatomies.

Reciprocating motion technology

Initially introduced in the 1950s, reciprocating motion involves a repetitive back-and-forth movement rather than continuous rotation. Early reciprocating systems, including M4 (SybronEndo, Orange, CA), Endo-Express (Essential Dental Systems, South Hackensack, NJ), and Endo-Eze (Ultradent Products, South Jordan, UT), employed equal clockwise (CW) and counterclockwise (CCW) motions to facilitate canal shaping. Although reciprocating files require more inward pressure than rotary files, their debris removal efficiency was initially limited. However, advancements in reciprocating technology have improved cutting efficiency and reduced instrument fatigue.

Fifth-generation Ni-Ti rotary files

The most recent innovations introduced an offset center of mass and/or rotation, enhancing flexibility, reducing taper lock, and allowing easier navigation of curved canals with minimal dentin removal. A notable example is the ProTaper Next system, which evolved from the widely used ProTaper Universal. ProTaper Next features an off-centered rectangular cross section, progressive-regressive tapers, and M-wire technology, improving flexibility and cyclic fatigue resistance while reducing the screw-in effect and torque demand [8].

The Mtwo Ni-Ti rotary system is another widely used endodontic file system, featuring an italic ‘S’-shaped cross section with two cutting blades. The flute depth and helical angle increase progressively from the tip to the handle, improving cutting efficiency and coronal debris removal. Additionally, the Mtwo system incorporates an increasing pitch length, which helps prevent threading and binding while reducing apical debris extrusion. The files also have a non-cutting tip and a negative rake angle, which contribute to conservative dentin preservation [9].

Despite these advancements, concerns remain regarding the impact of rotary instrumentation on root dentin integrity. Various studies have reported different degrees of dentinal microcrack formation associated with different Ni-Ti rotary systems. Instrument design, rotational speed, torque settings, and operator technique all play critical roles in determining the extent of stress-induced damage to root dentin. Therefore, it is essential to conduct comparative evaluations of different file systems to assess their respective advantages and risks in clinical practice.

This study aims to evaluate and compare transverse sections of root dentin after endodontic preparation using ProTaper Next (Dentsply Maillefer, Ballaigues, Switzerland) and Mtwo Ni-Ti (VDW, Munich, Germany) rotary file systems, with a particular focus on microcrack formation. By analyzing crack formation patterns associated with these two systems, the study seeks to contribute to a better understanding of their impact on dentin integrity and to provide clinical recommendations for safer and more effective endodontic instrumentation.

## Materials and methods

Study population and sample selection

A total of 15 orthodontic patients needed extraction of two identical single-rooted premolars located in the mandible during the study. For selection purposes, the department of orthodontics provided patients enrolled in treatment. The research only included premolars with single roots that showed canals on their preoperative periapical images. Exclusion criteria included teeth affected by either calcification of the canals or structural anomalies present in the tooth structure.

Experimental groups

A division occurred for the teeth after extraction according to which rotary file system was utilized for the canal preparations. The group of teeth on the left side of the arch received treatment with rotary file ProTaper Next provided by Dentsply Maillefer, while the group on the right side received treatment with rotary file Mtwo from VDW.

Materials and armamentarium

The study employed an Airotor handpiece (NSK, Tokyo, Japan) as well as rubber dam kit (GDC), Endo Access Bur (Dentsply Maillefer), K-files (#10, #15, #20) (Mani Inc., Utsunomiya, Japan), electronic apex locator (Root ZX Mini, J. Morita, Irvine, CA), Endoblock (Dentsply Maillefer), disposable syringes and 30-gauge disposable needles, X-Smart Torque Control Endomotor (Dentsply Maillefer), mandibular premolar extraction forceps, and low-speed saw machine (MS-10, Ducom, Bengaluru, India).

The clinical procedures utilized 3% sodium hypochlorite solution (Prevest DenPro Limited, Jammu, India), 0.9% normal saline (Ives Drugs Private Limited, Indore, India), 2% thymol solution (Pioneer Inorganics, New Delhi, India), 2% lignocaine with 1:200000 adrenaline (Cadila Pharmaceuticals, Ahmedabad, India) as well as EDTA gel (Prep Canal, Ammdent, Sahibzada Ajit Singh Nagar, India) and 17% aqueous EDTA liquid (Avue Prep+, Dental Avenue, Mumbai, India).

The research employed the following rotary file systems, as ProTaper Next files (Dentsply Maillefer) included four instruments that are X1 (17/.04), X2 (25/.06), X3 (30/.07), and X4 (40/.06) and Mtwo files (VDW): 20/.06, 25/.06, 30/.06, 35/.06, and 40/.06.

Clinical procedure

Tooth Preparation and Canal Instrumentation

All patients underwent anesthesia treatment with 2% lignocaine containing adrenaline at 1:200000 dosage while the dental professional placed a rubber dam. A dentist prepared the access cavity with Endo Access Bur according to a straight pattern design. Professionals used sodium hypochlorite solution 3% to clean the canal orifice before reaching the working length with electronic apex locator procedures, which were verified using radiographs according to Ingle’s method.

Root Canal Preparation

The participants in group 1 used ProTaper Next rotary files with EDTA as a lubricant to prepare the canal from file #20 to size 40 after performing initial preparation with a #20 K-file. The file system operated at 300 rpm speed with 2 Ncm torque settings. The working length protocol utilized the files X1 (17/.04), X2 (25/.06), X3 (30/.07), and X4 (40/.06) in sequence. The files operated in a circular motion while keeping light pressure on the apex during a brushing-like motion. Irrigation following each instrument exchange consisted of 2 mL of 3% sodium hypochlorite and subsequently included 5 mL of 17% EDTA before using 2 mL of sodium hypochlorite followed by a saline rinse. The group experimented extracted its teeth through trauma-free procedures then placed these teeth in 2% thymol solution storage.

Restoration of the Mtwo File System started with a #20 K-file before completing apical enlargement up to size 40 by using Mtwo rotary files, which received EDTA as a lubricant. The file operation used 300 rpm speed with 120 gcm torque. The instruments used for reaching working length comprised 20/.06, 25/.06, 30/.06, 35/.06, and 40/.06 in sequence. The medical workers applied the files through gentle brushing actions. The process of instrument change was followed by 2 mL 3% sodium hypochlorite irrigation, then completed with 5 mL 17% EDTA and 2 mL sodium hypochlorite and a rinse with saline. Dentists extracted the prepared teeth atraumatically and then submerged them in a 2% thymol solution.

Sectioning and microscopic examination

A low-speed saw machine (MS-10, Ducom) sectioned the extracted mandibular premolars at three specific distances from the apex, which were 3, 6, and 9 mm perpendicular to their long axis. Researchers placed the sectioned materials in 2% thymol during storage before proceeding with examinations.

Scanning electron microscopy (SEM) analysis

The evaluation of material specimens using a SEM (Zeiss Evo 18; Carl Zeiss AG, Oberkochen, Germany) under different magnification levels from 25× to 300× served to detect crack formation in sectioned samples. Two categories existed in the analysis of crack presence: no crack, where the dentin shows no signs of craze lines combined with microcracks or fractures that originate from within the canal lumen, and crack group, where the canal lumen generates microfractures as well as craze lines and fractures.

Statistical analysis

The research team conducted statistical examinations on recorded findings using appropriate tests to evaluate differences in crack development based on the two rotary file options. All analytical methods together with utilized software appear in the results section.

## Results

Overall crack formation

Crack formation was observed in both groups following root canal preparation. The number of samples exhibiting cracks versus those without cracks is summarized in Table 1.

**Table 1:** Number of samples with and without cracks χ²: chi-square test; df: degree of freedom A p-value of <0.05 is considered statistically significant.

Group	Samples with cracks (n = 15)	Samples without cracks (n = 15)	Chi-square test (χ²)	df	p-value
ProTaper Next	12 (80%)	3 (20%)	0.240	1	0.624
Mtwo	13 (86.66%)	2 (13.33%)			

**Figure 1:**
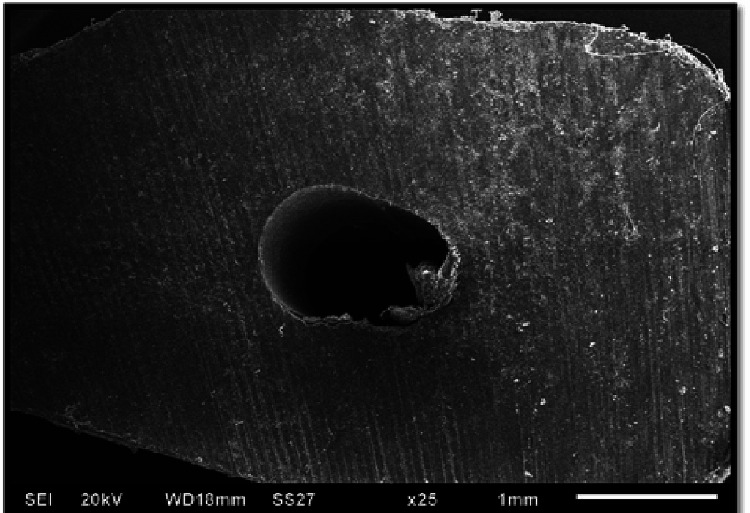
Sample showing no crack at 25× magnification under a scanning electron microscope

**Figure 2:**
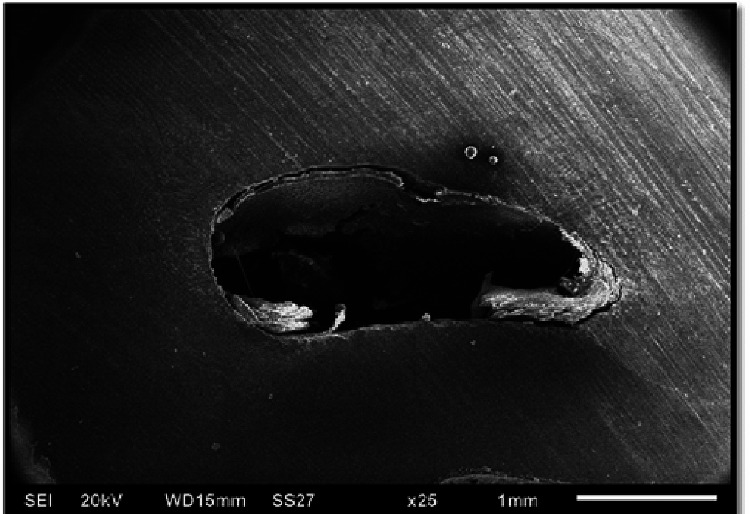
Sample showing crack at 25× magnification under a scanning electron microscope

Statistical analysis using the chi-square test showed no significant difference between the two groups (χ² = 0.240; df = 1; p = 0.624). A p-value of <0.05 is considered statistically significant (Figures 1, 2).

Crack formation at different root levels

Crack formation was further analyzed at coronal (9 mm), middle (6 mm), and apical (3 mm) levels for both groups, as shown in Table 2.

**Table 2:** Number of samples with cracks at different root levels A p-value of <0.05 is considered statistically significant.

Group	Coronal (9 mm)	Middle (6 mm)	Apical (3 mm)
ProTaper Next	8 (53.3%)	6 (40%)	2 (13.3%)
Mtwo	10 (66.7%)	10 (66.7%)	7 (46.7%)
p-value	0.456	0.143	0.046

The chi-square test revealed no statistically significant differences at the coronal (p = 0.456) and middle (p = 0.143) levels. However, at the apical level, Mtwo files resulted in significantly more cracks than ProTaper Next (p = 0.046).

Both ProTaper Next and Mtwo rotary files caused crack formation in root dentin. ProTaper Next files resulted in fewer cracks compared to Mtwo files, although the difference was not statistically significant (p > 0.05). Crack formation was most prominent at the coronal third, followed by the middle and apical thirds in both groups. The Mtwo group exhibited more cracks at the coronal and middle thirds than the ProTaper Next group; however, this difference was not statistically significant (p > 0.05). At the apical third, Mtwo rotary files caused significantly more cracks compared to ProTaper Next files (p < 0.05). These findings suggest that both rotary file systems contribute to crack formation, with Mtwo exhibiting a higher tendency, particularly in the apical region.

## Discussion

Biomechanical preparation plays a critical role in achieving endodontic success. It allows for effective bacterial elimination, debris removal, or the facilitation of three-dimensional obturation of the root canal system. Canal shaping is essential to provide sufficient space for application in irrigating solutions and medicaments, contributing to effective disinfection [10]. The goal of preparation is to achieve a uniformly tapered funnel shape, which increases in diameter from the apical end to the orifice.

Various methods can be employed for root canal preparation, including manual preparation, rotary instrumentation, sonic and ultrasonic systems, laser use, and non-instrumentation techniques. Traditionally, stainless steel hand files were utilized, although their disadvantages, such as increased time consumption, suboptimal cleanliness, and a higher incidence of procedural errors, prompted the introduction of Ni-Ti instruments in the 1990s. Ni-Ti instruments, with their lower elastic modulus and broader elastic working range compared to stainless steel, have since become the preferred choice for root canal preparation. Advantages of Ni-Ti rotary files over stainless steel instruments include superior cleanliness, better shaping, reduced canal straightening, fewer apical transportation, and a lower risk of perforations [11]. These instruments, due to their flexibility, have allowed for more efficient and error-free root canal preparation.

Ni-Ti rotary instruments offer additional advantages, such as a reduced risk of fractures and fewer procedural errors compared to stainless steel instruments. The flexibility of Ni-Ti, particularly in the form of M-wire alloy, contributes to enhanced flexibility and reduced stress exerted on the dentinal walls, which may lead to fewer cracks [12]. The design of Ni-Ti files also plays a significant role in reducing the forces applied to the dentin, thereby minimizing the risk of dentinal defects [11]. In this study, two different Ni-Ti rotary files, ProTaper Next (Dentsply Maillefer) and Mtwo (VDW), were compared to assess their impact on the occurrence of dentinal cracks following root canal preparation. Both file systems caused cracks, although ProTaper Next files were associated with fewer cracks than Mtwo. This difference, however, was statistically non-significant (p > 0.05). More cracks were observed in the coronal third of the root canal in both groups, which is consistent with previous studies indicating higher stress levels in the coronal portion of the root canal [13].

Statistically, Mtwo files caused significantly more cracks in the apical third compared to ProTaper Next files (p < 0.05). This finding may be attributed to the distinct design differences between the two file systems. ProTaper Next utilizes an off-centered rectangular cross section, which reduces contact with the canal wall, consequently decreasing stress concentrations and the risk of crack formation [11]. In contrast, Mtwo files, with their S-shaped cross section, tend to generate more stress, leading to a higher incidence of dentinal defects [14,15]. Crack formation at the apical third of the root canal was significantly more common with Mtwo files. Stresses generated by rotary instrumentation, particularly near the tip of the instrument, contribute to the development of cracks in this region [16,17]. These findings emphasize the need for clinicians to consider the design and flexibility of rotary instruments when selecting tools for root canal preparation, as excessive stress can lead to crack formation and, eventually, root fracture.

This study highlights the potential for Ni-Ti rotary files to cause cracks in root dentin, regardless of file design. However, the results suggest that ProTaper Next files, with their innovative design, exert less force on the root canal walls and cause fewer cracks compared to Mtwo files. While this difference was statistically non-significant, it may still have clinical relevance. Cracks, once initiated, can propagate and lead to root fractures over time, posing a significant risk to the longevity of the treated tooth. Therefore, clinicians should select rotary files that minimize the risk of dentinal defects while ensuring effective cleaning or shaping of the root canal system.

## Conclusions

The findings of the present study demonstrate that both ProTaper Next and Mtwo rotary file systems induce dentinal cracks following root canal preparation. However, the ProTaper Next system exhibited a lower incidence of crack formation compared to the Mtwo system, though this difference was not statistically significant (p > 0.05). Cracks were predominantly observed in the coronal third of the root canal in both groups, with fewer cracks noted in the middle and apical thirds. Additionally, the Mtwo file system resulted in a higher number of cracks in the coronal and middle thirds than ProTaper Next; however, this difference was also not statistically significant (p > 0.05). A statistically significant difference (p < 0.05) was observed in the apical third, where the Mtwo rotary files induced a greater number of cracks compared to ProTaper Next files. These results suggest that both file systems contribute to dentinal microcrack formation, but ProTaper Next may be a more favorable option for preserving dentin integrity, particularly in the apical third, during root canal instrumentation.
